# Vulcanite

**Published:** 1877-01

**Authors:** 


### EDITORIAL, ETC.

*Vulcanite.*—In advocating hard rubber as a base for artificial teeth we simply confirm the convictions and conclusions arrived at from a practice of no small dimensions, running through a period of fourteen years; and in point of success, in regard to comfort to the wearer and manipulation of the material, second to none in the State.

Having worked in gold and silver for ten years previous to the advent of rubber, we, of course, had our doubts and fears, our prejudices and quibbles, when the new material came into vogue, but having a disposition to be progressive, we boldly ventured to the front, and became the pioneer in its introduction into the community in which we have practiced successfully for twenty-eight years. Our first practical plate of rubber was fitted into the mouth of the wife of the Mayor of our city, July 30th, 1862. This was followed by three others shortly after, and all these having worn rubber plates during these fourteen years, have never suffered the least inconvenience, but regarded them as near nature as they supposed artificial work could be made. We have a sample set which was made in the winter of 1858–'59, but our experience as a practical worker in rubber is from the former date; and running a considerable practice, taking in all kinds of work, and meeting every case that presented itself with more than ordinary success, we feel that we have some claims to an opinion, and can judge of the merits of a material which is not without its friends and its enemies, but which has produced more solid comfort than any other material ever used in artificial dentures.

A great deal has been said and written in regard to the poisonous effects of the rubber upon the mucous membrane of the mouth, its unpleasant odor, and various other objections of a serious character, to prevent its use and destroy it for dental purposes, but in our experience it has less objections, and possesses more advantages than any other material we have ever used, gold not excepted. The only objection we can file against it was that it was subject to a royalty which became rather burdensome, and which provoked rather serious disgust just when the "tax-gatherer" came around, but to our certain knowledge, at no other time since the first plate that we put in the mouth, have we felt as though we could do a day without it. We believe that it will pass muster in every scientific test that can be instituted as to its entire freedom of injury, and in regard to its integrity, durability, and capacity ; to be so moulded as to fit well and cling to the mouth, it has no parallel.

We have had a few cases where that raw appearance of the membrane presented itself after a plate had been worn a long time, but this occurs with gold, silver or celluloid. We have had cases where that hot sensation was experienced, but have found the same thing true of all metals, and very especially with celluloid. There is no objeetion offered to it that does not present itself in every other material in use.

It has been said that by reason of its easy manipulation it has driven science out of the laboratory, and reduced mechanical dentistry to a mere handicraft. We do not believe that this is the fact. It does not require such a fine artistic ingenuity to construct a first class plate from rubber as from gold, but this is only in the work Every other part of the operation, the fitting, the restoration of contour, the position of the teeth, the articulation, the artistic style and finish of the work, need the ingenuity, judgment and experience of the educated dentist just as much in rubber as in gold. We had as bad work in gold and silver, and as many quacks in those days as now, and perhaps the very men who make the above complaint were more in fault in producing such results, by pretending a superior virtue in practice, and handing rubber to incompetent assistants, or quack neighbours around the corner to do their work, instead of unbending their dignity and accommodating their magnificence to the logic of events and progression.

But how about license, and the Goodyear Vulcanite Company during the above recited experience? Have we been on good terms with that troublesome company all this time, or have we been subject to the same system of legal process, injunctions and demands for license fees as our other brethren of the profession? *We have.* We know the whole true inwardness of that system, and have felt the iron heel with crushing force. Our trouble in that direction, however, seems to have been as much from our own obstinacy, as from their determination to press their claims. This is no argument, however, against the merits of Vulcanite as a base. Its triumph is complete, its excellence cannot be denied. As for others, they may do as they prefer, but our acknowledged preference over everything else is Vulcanite.—*Ed. of Penna. Journal.*

